# A Bumpy Ride of Mycobacterial Phagosome Maturation: Roleplay of Coronin1 Through Cofilin1 and cAMP

**DOI:** 10.3389/fimmu.2021.687044

**Published:** 2021-09-23

**Authors:** Saradindu Saha, Arnab Hazra, Debika Ghatak, Ajay Vir Singh, Sadhana Roy, Somdeb BoseDasgupta

**Affiliations:** ^1^ Molecular Immunology and Cellular Microbiology Laboratory, Department of Biotechnology, Indian Institute of Technology Kharagpur, Kharagpur, India; ^2^ Department of Microbiology and Molecular Biology, ICMR-National JALMA Institute of Leprosy and Other Mycobacterial Diseases, Agra, India

**Keywords:** mycobacteria, BMDM, Coronin1, cAMP, Cofilin1

## Abstract

Phagosome-lysosome fusion in innate immune cells like macrophages and neutrophils marshal an essential role in eliminating intracellular microorganisms. In microbe-challenged macrophages, phagosome-lysosome fusion occurs 4 to 6 h after the phagocytic uptake of the microbe. However, live pathogenic mycobacteria hinder the transfer of phagosomes to lysosomes, up to 20 h post-phagocytic uptake. This period is required to evade pro-inflammatory response and upregulate the acid-stress tolerant proteins. The exact sequence of events through which mycobacteria retards phagolysosome formation remains an enigma. The macrophage coat protein Coronin1(Cor1) is recruited and retained by mycobacteria on the phagosome membrane to retard its maturation by hindering the access of phagosome maturation factors. Mycobacteria-infected macrophages exhibit an increased cAMP level, and based on receptor stimulus, Cor1 expressing cells show a higher level of cAMP than non-Cor1 expressing cells. Here we have shown that infection of bone marrow-derived macrophages with H37Rv causes a Cor1 dependent rise of intracellular cAMP levels at the vicinity of the phagosomes. This increased cAMP fuels cytoskeletal protein Cofilin1 to depolymerize F-actin around the mycobacteria-containing phagosome. Owing to reduced F-actin levels, the movement of the phagosome toward the lysosomes is hindered, thus contributing to the retarded phagosome maturation process. Additionally, Cor1 mediated upregulation of Cofilin1 also contributes to the prevention of phagosomal acidification, which further aids in the retardation of phagosome maturation. Overall, our study provides first-hand information on Cor1 mediated retardation of phagosome maturation, which can be utilized in developing novel peptidomimetics as part of host-directed therapeutics against tuberculosis.

## Introduction

Tuberculosis (TB) caused by *Mycobacterium tuberculosis* (*M. tb*) affects a quarter of the globe and has mortality over 1.5 million every year (1). Lack of an environmental reservoir causes *M.tb* to be an obligate intracellular parasite that predominantly chooses alveolar macrophages as its host and evades the host immune system. A significant setback in tuberculosis treatment is the emergence of extreme and total drug-resistant *M. tb* arising out of unregulated and discontinuous use of anti-mycobacterial in socioeconomically backward populations (2). Host-directed therapeutics are coming of age. Therefore, the need of the hour is to identify new drug targets for which one must comprehend the molecular intricacies between the host defense system and mycobacterium. Mycobacterial pathogenesis involves the secretion of several virulence factors (PknG, SapM, PtpA, Eis) and the hijacking of host factors (Coronin1, Calcineurin, SOCS1, CISH, LRG47, Gbp5) to establish its niche within the macrophages (3). Evolutionarily, Coronins are a family of actin-binding proteins that regulate several cellular functions such as cell migration, cytokinesis, phagocytosis, cytoskeletal reprogramming, etc. (4, 5).

Cor1 is a 57-kDa, trimeric protein in mammals, explicitly expressed in the hematopoietic cells and brain. The interaction of Cor1 with F-actin has been established, but whether this interaction has any role in mycobacterial pathogenesis remains to be elucidated (6). Cofilin1 is another protein, which is activated through dephosphorylation followed by F-actin depolymerization by cooperatively binding along the sides of actin filaments and inducing conformational changes in filament structure (7). Cor1 is known to activate Cofilin1 by activating the phosphatase Slingshot and thereby affecting actin dynamics (8). Deletion of Cor1 affects actin reorganization dynamics, thus influencing phagocytosis (9). Trimeric Cor1 being a cortical protein is recruited and retained by live pathogenic mycobacteria and it hinders phagolysosome formation (6). Knockout of Cor1 does not affect phagocytosis, but mycobacteria-containing phagosomes in these cells are rapidly acidified and fuse with lysosomes. Macrophage Cor1 gets upregulated in active TB patients (10), while ectopic Cor1 expression in Cor1 non-expressing cells has increased intracellular cAMP levels (11). Mycobacterial infection of macrophages raises the intracellular cAMP levels, which then impacts pathogenesis through yet unknown mechanisms. Therefore, it remains to be revealed whether Cor1 mediated activation of Cofilin1 and Cor1 mediated increase in threshold levels of cAMP are two different events or part of an interlinked signaling cascade promoting mycobacterial pathogenesis.

When immortalized bone-marrow-derived macrophages (BMDM) were infected with live mycobacteria, the intracellular cAMP levels were raised, but the same did not occur in Cor1 knockout macrophages. A mycobacterial infection-induced nascent overexpression of Cor1 was also observed, which then led to the activation of Cofilin1, which then increasingly depolymerized F-actin at the vicinity of the phagosomes. Consequently, phagosomal acidification was hindered, thus hindering phagosome-lysosome fusion. Conversely, Cor1 knockout macrophages failed to activate Cofilin1 upon mycobacterial infection, thus causing F-actin accumulation at the phagosomes that then promoted its acidification. For the first time, this study elucidates the mechanism of Cor1 mediated hindrance to phagosome maturation upon mycobacterial infection. We ascribe that mycobacterial infection causes Cor1 mediated elevation of intracellular cAMP, followed by activation of Cofilin1 and thereafter depolymerization of F-actin to hinder phagosomal acidification and thus its maturation.

## Materials and Methods

### Cell Lines

Immortalized murine BMDM (WT and Cor1^-/-^) as described in Reference 12 were used in this study unless otherwise stated. All macrophages were maintained in DMEM (Sigma-Aldrich) supplemented with 10% heat-inactivated FBS (Gibco) at 37°C in a humidified incubator with 5% CO_2_. Cells were microscopically checked after every 24 hours and routinely split into new cultures when they reach 70% confluence.

### Mycobacterial Culture

*M. tb* H37Rv, *M. bovis BCG* (*BCG*), and *M. smeg* were grown on 7H9 liquid medium (Sigma) containing 0.08% Tween 80 and supplemented with 10% OADC enrichment. Parallel sets of mycobacterial cultures were maintained, and subcultures were given from those with little to no mycobacterial clumps. *M. tb* H37Rv cultures were carried out in a biosafety level 3 facility of National Jalma Institute of Leprosy and Other Mycobacterial Diseases (NJILOMD), Agra, and National Institute for Research on Tuberculosis (NIRT), Chennai. All experiments with *M. tb* were carried out according to institutional guidelines of NJILOMD and NIRT. *M. bovis BCG* and *M. smeg* were cultured inside biosafety class 2 type A2 cabinets at IIT Kharagpur as per the institutional guidelines. Dead mycobacteria were prepared by heating mycobacteria-containing cultures at 65°C for 30 min followed by washing and resuspension in DMEM containing 2% FBS.

### Reagents

Competitive cAMP-ELISA kits were procured from Cayman, rabbit monoclonal anti-LAMP1, anti-EEA1, and anti-Coronin1 antibody was from cell signaling technology (CST), mouse monoclonal anti-Cofilin1 antibody, rabbit monoclonal LAMP1, and rabbit monoclonal anti-phospho Cofilin1 antibody was from Sigma-Aldrich, and anti-cAMP antibody was from Santa Cruz Biotechnology. Mouse monoclonal anti-αtubulin antibody was from Novus Biologicals. Secondary antibodies conjugated with fluorophores AlexaFlour488 and 568 were from Life Technologies, DRAQ5 from CST. KH7, Rolipram, Slingshot inhibitor D3 were from Sigma-Aldrich. Protease inhibitor cocktail and phosphatase inhibitor were from Roche. 5x Bradford Reagent was from Bio-Rad, Supersignal West Pico chemiluminescent reagent, and ProLong Gold Antifade was from Life Technologies.

### Isolation of Total RNA, Preparation of cDNA, and Real-Time PCR Analysis

Total RNA was isolated from untreated or different inhibitor treated BMDM with or without mycobacterial infection using Roche Total RNA isolation kit as per manufacturers protocol. The quality of total RNA was analyzed using a microvolume spectrophotometer to 260/280 ratio. First-strand cDNA synthesis was carried out using oligo dT (Roche) and Superscript II RT (Thermo) as per manufacturers protocol. PCR amplification was initially standardized before real-time analysis using the sense and antisense primers TCGGACC TGTTCCAGGAGGA CTGGGCTCTGGTGTAGC TCTT for Cor1 and CATCACTGCCACCCAGAAGACTG and ATGCCA GTGAGCTTCCCGTTCAG for GAPDH, respectively. Next, real-time PCR was carried out using BioRad SSO advanced master mix and in a BioRad CFX96 system. Relative mRNA expression was quantified using the ΔΔCt method (12).

To analyze Th1/Th2 response inside BMDM macrophages upon infection with non-pathogenic *M. smegmatis* or pathogenic *M. tb (H37Rv)* at 0-h and 3-h chase times, the cells were pelleted, lysed, and total RNA was prepared and cDNA generated as described previously. Next, sense and antisense oligonucleotides for TNFa (GGTGCCTATGTCTCAGCCTCTT and GCCATAG AACTGATGAGAGGGAG), IL-10 (CGGGAAGACAA TAACTGCACCC and CGGTTAGCAGTATGTTGTCCAGC), iNOS_2_ (GAGACAGGGAAGTCTGAAGCAC and CCAGC AGTAGTTGCTCCTCTTC), and Arginase1 (AACACTCC CCTGACAACCAG and CCAGCAGGTAGCTGAAGGTC) were used for qPCR analysis having GAPDH as the housekeeping gene and using BioRad SSO advanced master mix and in a BioRad CFX96 system. Relative mRNA expression was quantified using the ΔΔCt method. The fold change in expression over the GAPDH expression level was plotted. The experiments were carried out in triplicate with each sample set being in duplicate for each of the experiments.

### Mycobacterial Infection

Mycobacterial infection of BMDM macrophages was carried out as described elsewhere (13). Before infection, cells were plated in a 10-cm dish or 6-well plates at 2.2 ×10^6^ or 3×10^5^ cells, respectively. Log phase mycobacterial culture was taken in a 15-ml tube and centrifuged at 1000 g for 5 min to remove clumped mycobacteria. After that, the supernatant was taken in a fresh tube, centrifuged again at 4500 g for 5 min, followed by three washes using DMEM containing 2% FBS, and finally resuspended in it before adding it to BMDM cells and thereafter kept at 37°C incubator with 5% CO_2_ for 1 h. Next, mycobacteria were removed, and the cells were washed with DMEM containing 10% FBS and incubated similarly for the different time periods as indicated. Unless otherwise stated, an MOI of 1:20 was used in our experiments, since at this MOI we obtained appreciable viability as well as infectivity as compared to MOI 1:10 where cell viability was higher but infectivity was lower, while at MOI 1:40 the infectivity was higher but cell viability was lower (**Supplementary Figure 1A**). Likewise, measurements of cAMP levels showed that for different MOI that were used, cells where an MOI of 1:20 was used for infection appropriate levels of cAMP were observed, which also increased with time of infection (**Supplementary Figure 1B**).

### Cloning and Transfection

Total RNA isolated from wildtype BMDM cells was used to generate cDNA as described earlier. Using the sense and antisense primers AGGCGCGCCTATGAGCCGGCA GGTGGTTCG and CGGCTCGAGCTACTTGGCCTGAAC AGTCT, respectively, Coronin1 was amplified and cloned in the AscI and XhoI sites of pCMV6-entry. The purified pCMV6-entry-Cor1 plasmid was mixed with Cor1^-/-^ BMDM in Buffer E1 of the Neon Transfection System, and transfection was carried out as per manufacturers protocol using the 100-μl kit. The expression of Cor1 in these transfectants was further confirmed through immunoblot analysis (**Supplementary Figure 2**).

### Competitive ELISA for Measurement of Intracellular cAMP

Wildtype, Cor1^-/-^, or Cor1 transfected Cor1^-/-^ BMDM cells (5×10^5^ cells/well of 6-well plates) were seeded for 24 h, after which they were either left untreated or pretreated with KH7 or Rolipram for 2 h. These cells were either kept uninfected or infected with *M. tb* (H37Rv), *M. bovis* BCG, or *M. smegmatis (M. smeg)* at different MOI (1:10, 1:20, 1:40) and chased for different time periods as indicated. After this, cells were washed with PBS three times to remove extracellular bacteria. After that, cells were lysed, and cAMP ELISA was carried out as per manufacturer’s protocol (Cayman). The indicated is an average of three independent experiments.

### Immunoblot Analysis

BMDM cells seeded at 5×10^5^ cells/well of a 6-well plate for 24 h were inhibitor-treated and thereafter infected with mycobacteria at MOI of 1:20 for different time periods as indicated. In one set of experiments, macrophages were pretreated with MG132 (5uM) for 1 h and infected with *M. tb* in the presence of the same concentration of MG132 for indicated time points and thereafter lysed and immunoblotted. Differentially treated and infected macrophages were next washed with ice-cold 1X PBS and subsequently incubated with RIPA lysis buffer containing protease inhibitor cocktail for 20 min on ice. The lysed cells were centrifuged at 16000 g for 20 min at 4°C, and the supernatant was taken in a new tube. Protein estimation was done, and equal amounts (30 μg) of protein corresponding to each sample were electrophoresed in 10% SDS-PAGE, transferred to PVDF membrane, blocked, and immunoblotted with different antibodies as indicated. Primary antibodies of different dilutions as standardized in 5% FBS containing PBS were used. The membranes were washed with PBS-T three times before incubation of species-specific secondary antibodies. The membranes were thereafter rewashed three times with PBS-T, followed by developing the membranes using Supersignal WestPico chemiluminescent reagent (Thermo) and analyzed under a LAS500 imager (GE). The best image out of three independent experiments has been represented, while densitometry analysis was carried out for the three experiments and the average data was plotted. The phospho-Cofilin1 specific densitometry values were generated after normalization with respective total Cofilin1 protein intensity.

### Immunofluorescence Microscopy

BMDM cells were seeded on Teflon-coated 10-well chamber slides for 2 h before mycobacterial infection at an MOI of 1:20 was carried out. Cells were incubated with mycobacteria for 60 min followed by a chase period as indicated. These infected cells were fixed using 4% paraformaldehyde for 15 min followed by permeabilization with 0.02% saponin (Sigma-Aldrich) and then blocked using 5% FBS in PBS for 1 h. After that, incubation in primary antibody, diluted in 5% FBS containing PBS, was carried out for 4 h, washed with PBS, incubated with respective AlexaFluor conjugated secondary antibody for 1 h, and washed again with PBS. The cells were counterstained with DRAQ5 (CST) when required to stain the nucleus. For staining mycobacteria, rabbit anti-mycobacteria antibody and AF488 conjugated donkey anti-rabbit were used. For labelling LAMP1 or LAMP2 and Cor1, rat anti-LAMP1 or LAMP2 and goat anti-Cor1 were used followed by counterstaining with AF568 conjugated donkey anti-rat and AF647 conjugated donkey anti-goat antibodies, respectively. The cells were mounted using ProLong Gold Antifade reagent and imaged under an FV3000 confocal microscope (Olympus).

### Measurement of Phagosomal Acidification Upon Mycobacterial Infection

For phagosomal pH measurement, mycobacteria were double-stained sequentially with 25 mM pH-sensitive pHrodo succinimidyl ester (Invitrogen) for 60 min at 37°C and 20 μg/mL pH insensitive Alexa Fluor 488 carboxylic acid (Invitrogen) as per manufacturers protocol. Thereafter, stained mycobacteria were washed three times with DMEM and resuspended in DMEM containing 2% FBS. BMDM cells were next infected using these labeled mycobacteria at an MOI of 1:20 for indicated time points. Non-internalized mycobacteria were removed by washing with PBS. Next, pH calibration was carried out by incubating infected cells in 10 mM phospho-citrate buffer with predetermined pH of 5 to 8. Finally, infected cells were fixed with 4% paraformaldehyde, and phagosomal pH was measured by monitoring the change in fluorescence of pH-sensitive dye pHrodo in a Cytation5 multimode reader (BioTek) against the fluorescent Alexa Fluor 488 fluorescence. For calculation of phagosomal pH, mean fluorescence intensity ratio of pHrodo and Alexa Fluor 488 was considered. Three independent experiments were carried out, and the average data were plotted.

### Quantification of Nascent Coronin1 Upon Infection

BMDM cells were cultured in L-methionine-free DMEM medium containing 10% dialyzed FBS for 2 h to deplete intracellular methionine level. After that, L-AZA (4-Azido-L-homoalanine, ortho analog of L-methionine) was added to the medium and incubated for another 2 h before infection with mycobacteria at MOI of 1:20. The presence of L-AZA instead of methionine causes nascent expressed proteins to harbor L-AZA. After infection for indicated time points for labelling of nascent proteins, the cells were washed three times with PBS and lysed with ice-cold lysis buffer containing 1% Triton-X-100. The lysate was incubated with biotin-alkyne. By exploiting the “click reaction” between Azide and alkyne, all the nascent expressed proteins that had been L-AZA labelled were biotin-alkylated (14). These proteins were next precipitated with Streptavidin-agarose and, after that, electrophoresed and immunoblotted using anti-Cor1 or anti-b-tubulin antibodies and processed as described earlier.

### Quantification of Mycobacterial Degradation

Macrophages can degrade pathogens by rapidly transferring them to lysosomes through the fusion of the phagosome and lysosome. Pathogenic mycobacteria are known to hinder phagosome maturation and fusion with lysosome while non-pathogenic mycobacteria are rapidly transferred to the lysosomes and degraded. Hence, in the context of our study we correlated phagolysosome formation to mycobacterial degradation and therefore the extent of phagolysosomes formation quantitated based on colocalized green, fluorescent mycobacteria and red fluorescent LAMP1 or LAMP2 stained lysosomes was observed and represented as a percentage of infected cells that were counted (n=100). The experiments were done in triplicate and the averaged values were plotted.

### Quantification of Phagosome Associated F-Actin

Both WT and Cor1^-/-^ BMDM were infected with mycobacteria at an MOI of 1:20 for indicated time periods followed by treatment with 1% formaldehyde to crosslink all complexes as well as phagosome-associated F-actin. After that, the cells were lysed using a hypotonic lysis buffer and passed through a 22-gauge needle to disrupt the large membrane complexes. This lysate was layered on top of a discontinuous sucrose gradient, and the F-actin crosslinked, mycobacteria-containing phagosomes were isolated as described earlier (15). These phagosomes were further processed for F-actin solubilization and immunoblotting, as mentioned in Jayachandran et al. (16).

To stain intracellular F-actin, Alexa Fluor 488 conjugated phalloidin was used. In order to obtain the extent of F-actin polymerization around the mycobacteria-containing phagosomes, a fixed line (denoted in red) specific fluorescence intensity measurement was carried out both from infected WT and Cor1^-/-^ BMDM. Fluorescence intensity values corresponding to two fixed lines were recorded from each image (n=50) and thereafter these recorded arbitrary fluorescence units were averaged and plotted for WT and Cor1^-/-^ either untreated or pretreated with slingshot inhibitor D3 prior to infection with *M. tb*.

### Transmission Electron Microscopy

Wild type and Cor1^-/-^ BMDM cells were infected with mycobacteria having an MOI of 1:20 for 2 h. After that, the cells were fixed in 2% glutaraldehyde in 0.2 M sodium cacodylate buffer containing 0.12 M sucrose for 1 h at 41°C. After washing, the cells were post-fixed in osmium tetroxide (1.5%, w/v) and stained in 0.5% uranyl acetate. Next, dehydration was carried out in ethanol, clearing in propylene oxide, and embedding in Epon 812 was performed according to standard procedures (17). Sections were stained in 5% (w/v) uranyl acetate and 0.4% (w/v) lead citrate. These sections were taken on copper grids by the floatation method.

Labelling was performed on the section by floating the grids (section side down) on droplets of labelling reagent on Parafilm M inside a humid chamber. Blocking was done with 0.5% BSA containing PBS for 5 min, followed by incubation with biotinylated-phalloidin for 1 h at RT. Next, the grids were washed with blocking buffer 6 times with 2 min for each wash, followed by incubation with streptavidin colloidal gold conjugate for 40 min at RT (18). The grids were rewashed 4 times with 2 min for each wash using blocking buffer. The grids were thereafter inserted into a Zeiss Orion TEM and visualized. In case of immunogold transmission electron microscopy, gold nanoparticle labelled target proteins appear as dense dots against the background of stained cells.

### Statistical Analysis

Results were analyzed with one-way ANOVA and *post hoc* multiple comparison test (Tukey HSD). Data are represented as mean ± SEM. p ≤ 0.05 was statistically significant. Significance was shown as follows: *p ≤ 0.05, **p ≤ 0.01, NS, non significant, graph plotted and statistical significance were shown using Origin8 and GraphPad Prism 5 software.

## Results

### Mycobacteria Infected Macrophages Exhibit an Increased Level of cAMP in Comparison to Uninfected Macrophages

**Figure 1:**
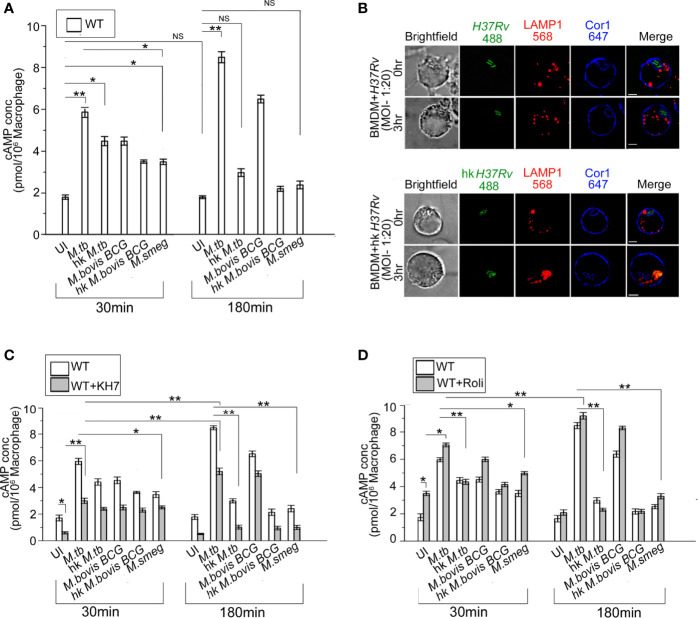
**(A)** Intracellular cAMP concentration in uninfected and live *M. tb*, *M. bovis BCG*, *M. smeg*, heat-killed *M. tb*, and heat-killed *M. bovis BCG* infected BMDM after the indicated time points using competitive ELISA. **(B)** Immunofluorescence analysis of mycobacteria-infected macrophages. WT-BMDM infected with live *M. tb* or hk *M. tb* for indicated time points were stained for mycobacteria (green), lysosomes (LAMP1 in red), and Cor1 (blue). In the case of live *M. tb-*infected macrophages Cor1, recruited around phagosomes at 0-h time point was retained even after 3 h of infection, while for hk *M. tb*-infected macrophages Cor1 was not retained after 3 h of infection (scale:10 μm). **(C, D)** Competitive ELISA-based measurement of intracellular cAMP concentration in uninfected or live *M. tb*, *M. bovis BCG*, *M. smeg*, heat-killed *M. tb*, and heat-killed *M. bovis BCG* infected BMDM for the indicated time points without and after pretreatment with **(C)** adenylate cyclase inhibitor KH7 or **(D)** phosphodiesterase inhibitor Rolipram (n = 3). Data represents mean ± SEM; *p < 0.05, **p < 0.01, NS, non-significant.

Increased intracellular cAMP is known to suppress the innate immune response and hinder phagosome maturation by interfering with phagosomal actin assembly (19–21). Nevertheless, whether this rise of intra-macrophage cAMP upon mycobacterial infection was caused by mycobacteria alone (22) or if macrophage signaling plays a role remained undeciphered. To investigate this, we infected BMDM with mycobacteria and thereafter measured intracellular cAMP by competitive ELISA. It was observed for uninfected macrophages that there was no rise in cAMP levels with time but when these BMDM were infected with *M. tb* there was an initial burst of cAMP at 30 min, which increased significantly after 180 min of infection (**Figure 1A**). A similar observation was also seen in the case of *M. bovis BCG*. When BMDM were infected with heat-killed mycobacteria, the initial level of cAMP at 30 min was slightly higher than the uninfected control, but it declined to levels similar to uninfected BMDM after 180 min of infection. This initial surge of cAMP after 30 min was generated by macrophages alone as the infecting mycobacteria were heat-killed, thus indicating that macrophages do contribute toward the increased level of intracellular cAMP upon mycobacterial infection. Next, when we carried out infection with non-pathogenic *M. smeg*, a similar initial surge of cAMP at 30 min was observed and the levels were between that of heat-killed mycobacteria and live pathogenic mycobacteria, since *M. smeg* although being non-pathogenic does contribute partly to the intracellular cAMP level. Non-pathogenic, *M. smeg* containing phagosomes fuse with lysosomes by 180 min post-infection. Hence, the cAMP levels also fall considerably at this time.

Compared to live *M. tb* or *M. bovis BCG*, heat-killed *M. tb* or *M. smeg* fails to retain the Cor1 coat on the phagosome membrane (23). When we infected BMDM with live pathogenic and heat-killed *M. tb*, it was observed that both live and heat-killed *M. tb* had Cor1 recruited to the respective phagosomes at the 0-h time point but only live *M. tb* containing phagosomes could retain the Cor1 coat after 3 h of infection and heat-killed *M. tb* containing phagosomes fused with lysosomes as it failed to retain the Cor1 coat around the phagosome (**Figure 1B**). Therefore, the decrease of cAMP with increasing time of infection in the case of heat-killed pathogenic mycobacteria or non-pathogenic mycobacteria could be attributed to its failure in retaining the Cor1 coat on the phagosome membrane because Cor1 has earlier been shown to increase cAMP levels (11).

Next, we pre-incubated cells with the adenylate cyclase antagonist, KH7, and thereafter measured cAMP levels with or without mycobacterial infection. The cAMP levels of KH7 pretreated BMDM at 30 min were lower than that in untreated cells (**Figure 1C**). Although infection of KH7 pretreated BMDM with *M. tb* and *M. bovis BCG* exhibited a rise of cAMP levels compared to uninfected macrophages at 30 min, the levels were much lower than that observed in untreated cells. A similar trend was observed in the case of infection with heat-killed mycobacteria or non-pathogenic *M. smeg* (**Figure 1C**). After 180 min of infection of KH7 pretreated BMDM with *M. tb* and *M. bovis BCG*, the cAMP values did increase due to gradual reactivation of inhibited ACs with time, but the levels were not sufficient in preventing phagolysosome formation (**Supplementary Figure 1C**). Infection with heat-killed mycobacteria or *M. smeg* at 30 min of infection exhibited reduced cAMP levels compared to KH7 untreated cells but infected BMDM, and the difference remained even after 180 min of infection. This can be attributed to the lack of mycobacterial contribution toward an increase of cAMP both for heat-killed mycobacteria or non-pathogenic *M. smeg* (24–26). Concomitant to decreased levels of cAMP after 180 min of infection, the heat-killed mycobacteria or *M. smeg* containing phagosomes fused with the lysosomes (**Supplementary Figure 1C**). This could also be attributed to the failure in retaining the Cor1 coat on the phagosome membrane.

BMDM cells exhibit a higher level of cAMP in Rolipram treated cells owing to its inhibition of PDE4 (27), but the levels reduced after 3 h due to reactivation of PDE4 (**Figure 1D**). Infection of these Rolipram pretreated cells with *M. tb* or *M. bovis BCG* exhibited a higher level of cAMP at 30 min, which increases further after 3 h of infection (**Figure 1D**). Infection with heat-killed mycobacteria or non-pathogenic *M. smeg* exhibited cAMP levels slightly higher than that observed for Rolipram treated but uninfected cells at 30 min, and the cAMP level decreases considerably after 3 h of infection to levels lower than untreated levels for heat-killed mycobacteria but slightly higher in *M. smeg* infected cells (**Figure 1D**). But these levels were not sufficient to hinder phagolysosome formation (**Supplementary Figure 1C**). This fall in cAMP levels and concomitant phagolysosome formation could be attributed to the inability of heat-killed *M. tb* or *M. smeg* to retain the initial phagosome membrane-recruited Cor1. To ascertain this hypothesis, we carried out similar cAMP assays in Cor1 knockout BMDM.

Apart from LAMP1, LAMP2 is another lysosomal marker and sometimes a better determining factor in terms of phagolysosome formation. Hence, we carried out colocalization studies of mycobacteria with LAMP2 at indicated chase time points after infection with non-pathogenic *M. smeg* or pathogenic *M. tb.* Representative images as shown in **Supplementary Figure 1D** show that lysosomes are located close to mycobacteria-containing phagosomes at 0-h time point for *M. smeg* infected BMDM, and at 3 h phagosome-lysosome fusion has occurred in these cells. In contrast, for *M. tb* infected cells from 0-h timepoint itself the Cor1 scaffold around the phagosome is observed and it stays at the 3-h time point as well, thus hindering LAMP2 stained lysosomes to fuse with these phagosomes. To further analyze the role of AC inhibitor KH7 and PDE4 inhibitor Rolipram in the context of phagolysosome formation with respect to decrease or increase in the levels of intracellular cAMP, we pretreated cells with these inhibitors prior to infection with non-pathogenic *M. smeg* or pathogenic *M. bovis BCG* or *M. tb.* It was observed that phagolysosome formation majorly occurred in the case of *M. smeg* infection, and inhibition of PDE4 by Rolipram to increase intracellular cAMP to some extent could not prevent this phagolysosome formation (**Supplementary Figure 1E**). Infection with pathogenic mycobacteria by virtue of its ability to retain the Cor1 coat on the phagosome membrane could increase the cAMP levels and together with mycobacteria-generated cAMP could reach an above threshold level to hinder phagosome maturation. But KH7 treatment hindered the Cor1-induced macrophage cAMP production, thus reducing the intracellular cAMP level to be below the required threshold and hence causing the phagosomes to mature and fuse with lysosomes in the *M. bovis BCG* and *M. tb* infected macrophages.

### Coronin1 Knockout Macrophages Exhibit a Lower Level of cAMP

**Figure 2:**
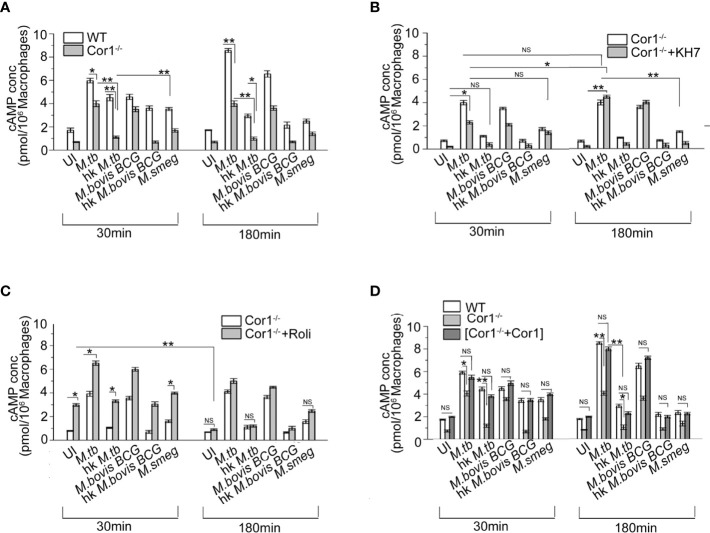
Competitive ELISA-based measurement of intracellular cAMP concentration in uninfected and live *M. tb*, live *M. bovis BCG*, live *M. smeg*, heat-killed *M. tb*, and heat-killed *M. bovis BCG* infected **(A)** WT and Cor1^-/-^ BMDM, **(B)** Cor1^-/-^ BMDM without or after pretreatment with adenylate cyclase inhibitor KH7, and **(C)** Cor1^-/-^ BMDM without or after pretreatment with phosphodiesterase inhibitor Roliparm or **(D)** transfected with pCMV6-Coronin1 for indicated time points (n = 3). Data represents mean ± SEM; *p < 0.05, **p < 0.01, NS, non-significant.

Cor1, being a cortical macrophage protein, gets automatically recruited to mycobacterial phagosomes (16), but only live pathogenic mycobacteria can retain it on the phagosomal membrane through its secretion of lipoamide dehydrogenase (23). Ectopic expression of Cor1 in Cor1 non-expressing cells is known to exhibit increased cAMP levels upon GPCR triggering (11). Results from **Figures 1A** **, C, D** show that apart from mycobacteria, macrophages also contribute to the increase in intracellular cAMP levels upon mycobacterial infection (24). Hence, infection of Cor1^-/-^ BMDM with live *M. tb* or *M. bovis BCG* resulted in cAMP levels that were lower than that of WT BMDM at 30 min. After 180 min of infection, Cor1^-/-^ BMDM did not exhibit any rise in cAMP levels while there was a significant rise in cAMP levels of WT-BMDM infected macrophages (**Figure 2A**). The difference in cAMP levels between WT and Cor1^-/-^ macrophages after 30 min of infection is indicative of the initial contribution of Cor1 toward cAMP production upon mycobacterial infection. The continued generation of cAMP owing to the presence of phagosome membrane bound Cor1 along with the mycobacteria-generated cAMP results in the high level of cAMP at 180 min post-infection. Thus, it was evident that the phagosome retained Cor1 contributes toward cAMP production with increasing time of infection and mycobacteria-generated cAMP only helps to keep the cAMP level at a higher threshold and static with increasing time of infection. Infection of Cor1^-/-^ BMDM with heat-killed mycobacteria did not produce any appreciable amount of cAMP as compared to infected WT-BMDM or live mycobacteria infected Cor1^-/-^ BMDM, and non-pathogenic *M. smeg* did not produce any appreciable amount of cAMP compared to infection of Cor1^-/-^ BMDM with live mycobacteria or with infection of WT-BMDM with heat killed *M. tb.* The difference in the cAMP levels between Cor1^-/-^ BMDM infected with live or heat-killed mycobacteria remained similar after 180 min of infection, but the difference in cAMP levels between WT and Cor1^-/-^ BMDM infected with heat-killed mycobacteria reduced after 180 min of infection. Similarly, infection of Cor1^-/-^ BMDM with non-pathogenic *M. smeg* after 30 min of infection exhibited a slightly lower level of cAMP compared to infection of WT-BMDM and the difference was non-significant after 180 min of infection.

Infection of KH7 pretreated Cor1^-/-^ BMDM exhibited higher levels of cAMP at 30 min compared to untreated and uninfected Cor1^-/-^ cells (**Figure 2B**), and this cAMP level increased considerably after 180 min of infection. The cAMP levels of untreated Cor1^-/-^BMDM when infected with live pathogenic mycobacteria did not exhibit any increase, but there was a significant rise in the cAMP levels from 30 to 180 min after infection, between KH7 pretreated Cor1^-/-^. This could be attributed to reduced initial level of cAMP in KH7 pretreated Cor1^-/-^ cells as compared to untreated Cor1^-/-^ and thereafter a gradual cAMP production by adenylate cyclase with time due to the absence of KH7. The cAMP levels in KH7 pretreated Cor1^-/-^ BMDM, upon infection with heat-killed mycobacteria, were similar to uninfected KH7 treated Cor1^-/-^ BMDM. Interestingly, KH7 pretreated Cor1^-/-^ BMDM when infected with *M. smeg* exhibited cAMP levels closer to KH7 pretreated Cor1^-/-^ BMDM infected with live *M. tb* or *M. bovis BCG* at 30 min, but the cAMP levels fell for the former and rose for the later after 180 min of infection. This indicated that the initial level of cAMP at 30 min was contributed through infection in general but *M. smeg* being non-pathogenic failed to contribute toward cAMP production, which in contrast was carried out by live *M. tb* or *M. bovis BCG*.

Next, when Cor1^-/-^ BMDM were treated with Rolipram, the cAMP level at 30 min was slightly elevated, and it decreased significantly after 180 min and became similar to untreated cells (**Figure 2C**). Since mycobacterial ACs generate an appreciable amount of cAMP, the live mycobacteria-infected macrophages in the Rolipram pretreated Cor1^-/-^ BMDM exhibit a higher amount of cAMP at 30 min compared to untreated but infected cells. The cAMP level falls thereafter and becomes closely similar to that of untreated but infected cells (**Figure 2C**). Similarly, significant difference exists between the cAMP levels of untreated and Rolipram treated Cor1^-/-^ BMDM after 30 min of infection with heat-killed mycobacteria or non-pathogenic *M. smeg*, but the cAMP levels become similar after 180 min of infection. Therefore, it is evident that the mycobacterial ACs, although capable of holding the cAMP level, are not enough to maintain it above the required threshold that is crucial for hindering phagosome maturation

Interestingly, when we expressed Cor1 inside Cor1^-/-^ BMDM (**Supplementary Figure 2**) and measured the cAMP level in these transfectants with and without mycobacterial infection, it was observed that the cAMP values were close to that obtained from wild type BMDM, with and without mycobacterial infection (**Figure 2D**). The time-wise trend of increase or decrease of cAMP levels between that of (Cor1^-/-^+Cor1) transfectant and Cor1^-/-^ BMDM were similar to that observed in **Figure 2A** between WT-BMDM and Cor1 BMDM. This indicated that the differences observed in Cor1^-/-^ were strictly due to the absence of Cor1-mediated cAMP production.

### Mycobacterial Infection Induced Nascent Up-Regulation of Macrophage Coronin1

**Figure 3:**
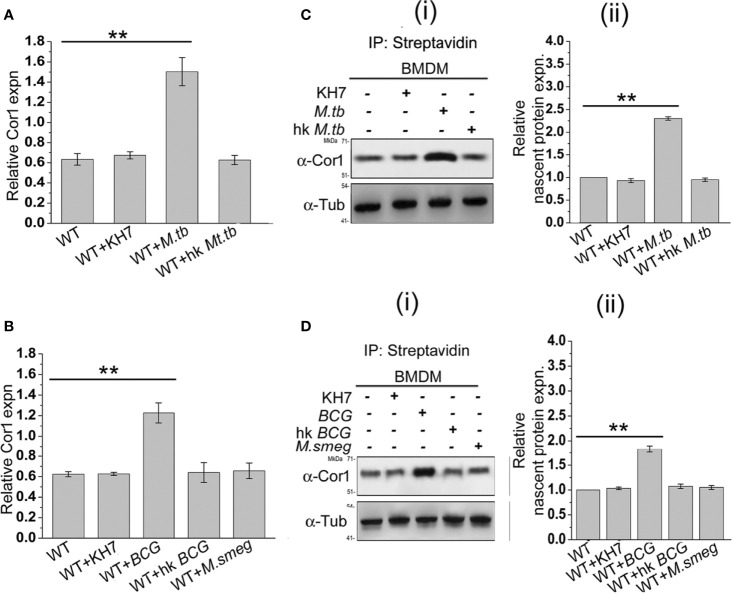
**(A)** Relative Cor1 expression against control b-tubulin expression in BMDM when kept uninfected, KH7 treated or **(A)** live *M. tb*, heat-killed *M. tb* infected or **(B)** live *M. bovis BCG*, heat-killed *M. bovis BCG*, or live *M. smeg* infected BMDM. Infection was carried out with MOI of 1:20 for 3 h (n = 3). **(C)** Nascent proteins labelled with L-AZA and thereafter biotinylated were immunoprecipitated using streptavidin=agarose and then immunoblotted with anti-Cor1. Immunoblots and their corresponding densitometric analysis were carried out for BMDM, which were either untreated or treated with KH7 or **(C)** (i), (ii) infected with live *M. tb*, heat-killed *M. tb*, or **(D**) (i), (ii) infected with live *M. bovis BCG*, heat-killed *M. bovis BCG*, or live *M. smeg*, respectively (n = 3). Data represents mean ± SEM; **p < 0.01.

From the previous experiments, it is evident that mycobacterial infection-induced increased cAMP inside the macrophages is partly produced by mycobacteria and partly by the phagosome recruited Cor1 and the rise in cAMP level is due to retention of Cor1 on the phagosome by pathogenic mycobacteria. To understand the mechanism of Cor1-mediated rise of intracellular cAMP, we tested the transcript level of Cor1 in *M. tb*, *M. bovis BCG*, or *M. smeg*-infected macrophages after 3 h of infection using qRT PCR. Both live *M. tb* and *M. bovis BCG* infected macrophages exhibit a ~2.4-fold and 2-fold increase in Cor1 mRNA expression after 3 h of infection compared to uninfected macrophages, while there was no notable change in Cor1 expression upon infection with heat-killed mycobacteria or with *M. smeg* (**Figures 3A** **, B**). Next, we wanted to see if this increased level of Cor1 transcripts is also reflected at the protein level. Surprisingly, immunoblotting of mycobacteria-infected macrophage lysates did not exhibit any significant change in expression of Cor1 (**Supplementary Figure 3A**).

We assumed that the subtle difference in Cor1 level upon infection could be masked due to the long half-life of the abundant level of existing Cor1 in the cell or part of Cor1 could exist in a state of dynamic equilibrium of expression and degradation, and the increased expression of Cor1 is required to replenish the continuously degrading Cor1. If Cor1 gets degraded continuously, the hint for the same would be obtained by immunoblotting mycobacteria-infected macrophages that had been pretreated and also in the presence of the proteasomal degradation inhibitor MG132. Such an experiment exhibited a gradual increase in the level of Cor1 above that of the constitutive gene control. Pre-incubation as well as the presence of MG132 inhibited the 26S proteasome thus hindering degradation of both Cor1 as well as the constitutive gene b-tubulin. If mycobacterial infection increased the nascent expression of Cor1, then inhibition of the 26S proteasome would be exhibited by a greater increase in Cor1 level over the increase observed for the constitutive b-tubulin. From **Supplementary Figure 3B** we observed that a similar result was obtained and the nascent expression of Cor1 over control was plotted in the densitometry analysis. We next decided to study the nascent changes in Cor1 expression upon infection, using a non-radioactive L-AZA mediated assay as described in the *Materials and Methods* section. From this experiment, the difference in the expression level of nascent Cor1 was evident in the case of live mycobacteria and not heat-killed mycobacteria-infected macrophages (**Figures 3C** **, D**). Therefore, it can be stated that macrophages infected with live pathogenic mycobacteria not only recruit and retain Cor1 onto the phagosomal membrane but also bring about a nascent increase in Cor1 expression. This nascent increase of Cor1 expression is required to replenish the decreasing level of Cor1 on the phagosome membrane and thereby initiate cAMP production. Thus, the cumulative effect of retained Cor1 and increased Cor1 expression contributes toward the rise in cAMP levels above threshold values required to hinder phagosome maturation.

From the above data it is evident that mycobacterial infection leads to nascent overexpression of Cor1, which is essential to maintain the macrophage-generated cAMP essential to reach a threshold level of cAMP along with mycobacterial cAMP to hinder phagosome maturation. Whether such nascent overexpression has any implication in the Th1/Th2 response of the macrophage, when pretreated with AC inhibitor KH7 or PDE4 inhibitor Rolipram prior to infection with *M. smeg* or *M. tb*, must be determined. As markers of Th1 or pro-inflammatory response, we chose to monitor TNFa and iNOS2 expression, while markers chosen for Th2 or anti-inflammatory response were IL-10 and Arginase1 (Arg1). Indeed, it was observed that infection with *M. smeg* had higher levels of TNFa and iNOS2, which is indicative of a pro-inflammatory Th1 response in the context of the infected macrophages while infection with *M. tb* exhibited a higher IL-10 and Arg1 levels, which is indicative of a Th2 or anti-inflammatory response in the context of the macrophages (**Supplementary Figure 4**). Interestingly, BMDMs pretreated with KH7 prior to *M. tb* infection shifted the balance from Th2 to Th1 response as evident from higher TNFa and iNOS2 levels in comparison to IL-10 and Arg1 levels. This could be the reason behind the phagosomal maturation and lysosomal fusion in these cells irrespective of infection with pathogenic *M. tb*. Although Rolipram is known to downregulate TNFa levels and dampen Th1 response (28), pretreatment of BMDM with Rolipram, although increasing the cAMP levels in *M. smeg* infected BMDMs, could not achieve a Th1 to Th2 switch. Hence, phagolysosome formation was not hindered in these cells. The inability of *M. smeg* to retain Cor1 on the phagosome could be playing a role as well. Therefore, we wanted to understand the role of Cor1-induced increased level of cAMP at the vicinity of the phagosome.

### Cofilin1 Is Activated by Mycobacterial Infection-Induced Overexpressed Coronin1

**Figure 4:**
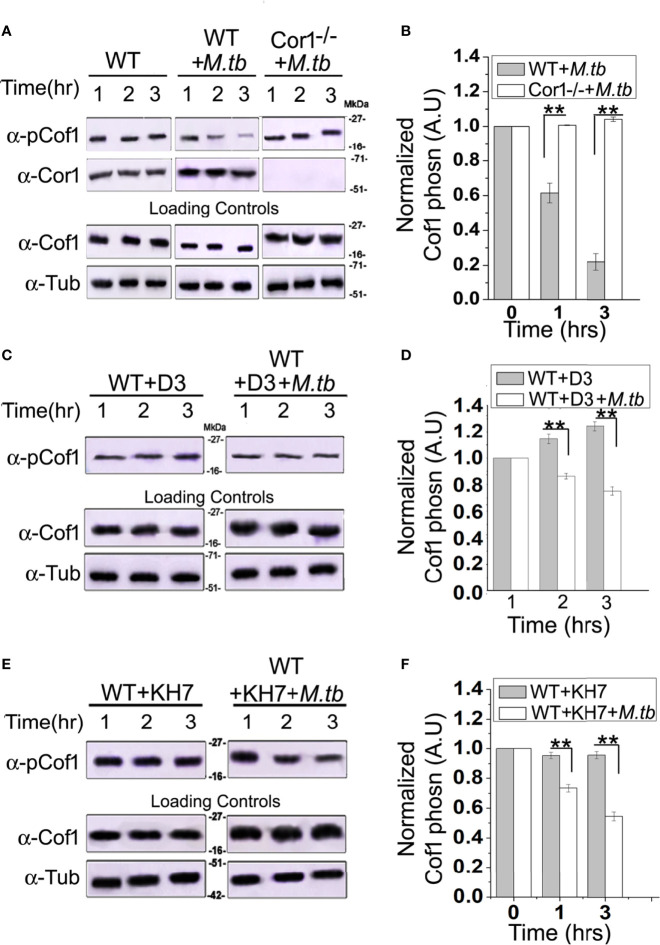
Immunoblot analysis **(A, C, E)** and its corresponding densitometric analysis **(B, D, F)** for Cofilin1 phosphorylation that were carried out in **(A)** untreated and uninfected BMDM (left panel), **(A, B)** untreated but *M. tb* infected WT (middle panel) or Cor1^-/-^ (right panel) BMDM, **(C, D)** D3 pretreated (left panel) or D3 pretreated and *M. tb* infected (right panel), **(E, F)** KH7 pretreated (left panel) or KH7 pretreated and *M. tb* infected wild-type BMDM, respectively (n = 3). Infections were carried out at an MOI of 1:20 for indicated time points. Immunoblots of Cofilin1 and b-tubulin served as loading control for these experiments. Data represents mean ± SEM; **p < 0.01.

The role of Cor1 is essential in hindering phagosome-lysosome fusion (29). Our data suggest that Cor1 has a significant contribution to the rise of cAMP inside macrophages upon mycobacterial infection. Previous literature suggests an increase in intracellular cAMP level activates the actin-binding protein Cofilin1 by dephosphorylating it (30). Ubiquitous Coronin2 is known to activate Cofilin1 by inducing its dephosphorylation through the engagement of the phosphatase Slingshot, and general activation of Cofilin1 occurs through its dephosphorylation by protein phosphatase 2A (8). Considering all these data, we hypothesized that mycobacterial infection-induced phagosome recruited Cor1 causes a rise in intracellular cAMP, which then activates a phosphatase to dephosphorylate Cofilin1 and thereby activate it. To address this possibility, we studied Cofilin1 dephosphorylation through immunoblotting. A continuous decrease in the level of phospho-Cof1 with increasing time of infection with *M. tb* was observed, where there was no decrease in phospho-Cof1 in the uninfected BMDM (**Figure 4A**). Densitometry analysis shows an ~80% decrease in the level of phospho-Cof1 after 3 h of macrophage infection with mycobacteria (**Figure 4B**). Contrary to this, no reduction of phosphor-Cof1 was observed in mycobacteria infected Cor1^-/-^ BMDM cells (**Figure 4A**), and densitometry analysis also shows the same (**Figure 4B**). Total Cof1 remains constant both in the case of mycobacteria-infected WT and Cor1^-/-^ BMDM cells, thus indicating that mycobacterial infection-induced activation of Cof1 occurs only in WT BMDM.

Next we pretreated wild type BMDM with Slingshot Inhibitor D3 and thereafter either kept them uninfected or infected them with *M. tb* for the indicated time points. Immunoblot analysis showed that the level of phosphor-Cof1 in uninfected but D3 pretreated macrophages remained constant. In contrast, D3 pretreated and infected macrophages exhibited only a negligible increase in infection time (**Figure 4C**), and the same is also observed in the densitometry analysis (**Figure 4D**). These results indicated that mycobacteria recruited Cor1 leads to the activation of the phosphatase, Slingshot, to dephosphorylate Cof1, thereby activating it. Pretreatment with Slingshot Inhibitor D3 prevented this dephosphorylation and thereby the activation of Cof1 upon infection.

Mycobacterial infection recruits and retains Cor1 on the phagosome membrane, contributing to the rise of intracellular cAMP. We next wanted to study whether this increased level of cAMP is required to activate Cof1. For this, we pretreated BMDM cells with adenylate cyclase inhibitor KH7 and then either kept them uninfected or infected them with mycobacteria for the indicated time periods. Immunoblot analysis showed that the KH7 treatment does not exhibit any change in the level of phosphor-Cof1. Simultaneously, there was an appreciable reduction in the extent of Cof1 dephosphorylation with time upon KH7 pretreatment before mycobacterial infection (**Figure 4E**), thus indicating that hindering cAMP production does impede the activation of Slingshot, which then reduces the extent of Cof1 dephosphorylation and thereby its activation. Densitometry analysis shows that after 3 h of mycobacterial infection, there is only an ~40% decrease in phospho-Cof1 level in KH7 pretreated cells compared to untreated cells where an ~80% decrease was observed (**Figure 4F**). These data indicate that the mycobacteria-containing phagosome retained Cor1 increases the intracellular cAMP level, leading to the activation of Slingshot, which then dephosphorylates Cof1 and activates it. Hence, next we wanted to study the consequence of Cof1 activation upon infection.

### Activated Cofilin1 Depolymerizes F-Actin at the Vicinity of Mycobacteria-Containing Phagosome

**Figure 5:**
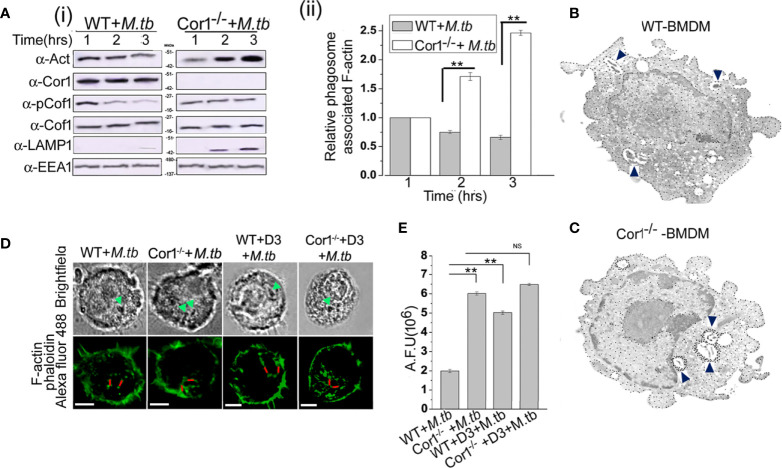
**(A)** (i) Immunoblot of F-actin, Cor1, pCof1, LAMP1, and EEA1 and (ii) densiometric analysis of F-actin, all crosslinked to isolated phagosomes from *M. tb* infected [**A** (i) left panel] wild type and [**A** (i) right panel] Cor1^-/-^ BMDM. Infection was carried out at MOI 1:20 for indicated time points. **(B, C)** Immunogold transmission electron microscopy for F-actin was carried out in *M. tb* infected **(B)** WT and **(C)** Cor1^-/-^ BMDM. Infection was carried out at MOI 1:20 for 90 min. Blue arrows indicate *M. tb*-containing phagosomes in both cell types with dense dots corresponding to F-actin around mycobacteria-containing phagosomes in Cor1^-/-^ BMDM. **(D)** Immunofluorescence analysis of F-actin (phalloidin-Alexa Fluor 488-green) around mycobacteria-containing phagosomes in *M. tb* infected WT (left panel) and Cor1^-/-^ (right panel) BMDM. Green arrows in the brightfield indicate mycobacteria-containing phagosomes, while the red line corresponds to the arbitrary fluorescence units (AFU) corresponding to polymerized F-actin around phagosomes (scale: 10 μm). **(E)** AFU values corresponding to the number of 50 cells were averaged and plotted. Representation for wild type or Cor1^-/-^ BMDM either untreated or pretreated with D3 and after that infected with *M. tb* at MOI of 1:20 for 90 min was carried out. Data represents mean ± SEM; **p < 0.01, NS, non-significant.

Fusion of mycobacteria-containing phagosomes with lysosomes requires F-actin tracks that guide the phagosome toward the lysosome (31). Activated Cof1 is known to associate with F-actin filaments and induce its depolymerization (19, 21). Hence, we wanted to check if Cor1 induced cAMP production upon mycobacterial infection results in activation of Cof1, which prevents phagosome lysosome fusion by causing F-actin depolymerization at the vicinity of the phagosomes. For this, we infected WT and Cor1^-/-^ BMDM with mycobacteria for the indicated time points, followed by crosslinking of cellular protein, lysis of these cells, phagosome isolation, and thereafter immunoblotting. The obtained results showed a slight decrease in F-actin level for the wild-type infected macrophages with increasing time of infection (**Figure 5A**). In contrast, in the case of Cor1^-/-^ macrophages, an increased amount of F-actin was observed on the phagosomes with increasing infection time. Since lack of Cor1^-/-^ failed to increase intracellular cAMP, Cof1 remains phosphorylated and at the vicinity of the phagosomes, thus getting crosslinked and isolated along with the phagosomes. This then allowed F-actin polymerization. The presence of Cor1 on the phagosomes of *M. tb* infected WT-BMDM resulted in an increased level of cAMP that then led to dephosphorylation of Cof1. Hence, the dephosphorylated form of Cof1 was isolated along with the phagosomes as the same could prevent actin polymerization. Interestingly, immunoblotting with lysosomal marker LAMP1 shows that only in Cor1^-/-^ macrophages phagolysosome fusion occurs due to increased F-actin polymerization at the vicinity of the phagosome.

This F-actin polymerization at the vicinity of the phagosome was further studied using immunogold transmission electron microscopy, where biotin-conjugated phalloidin was used to bind F-actin and gold nanoparticle conjugated streptavidin was used to label the F-actin that had bound by biotinylated phalloidin. In the case of immunogold transmission electron microscopy, gold nanoparticle-labelled target proteins appear as dense dots against the background of stained cells. Since we targeted F-actin, which is an abundant protein in the cell, there were numerous dense dots corresponding to intracellular, polymerized F-actin visible through immunogold electron microscopy. But an increased amount of dense dots corresponding to gold nanoparticle-labelled polymerized F-actin was observed at the vicinity of the mycobacteria-containing phagosome of Cor1^-/-^ BMDM (**Figure 5C**) as compared to mycobacteria-containing phagosomes inside WT BMDM (**Figure 5B**) after 2 h of infection. This clearly indicated that an appreciable amount of F-actin polymerization was occurring around the mycobacteria-containing phagosomes of Cor1^-/-^ macrophages, which was due to the failure of Cor1^-/-^ macrophages to increase intracellular level of cAMP, thus causing Cof1 to stay phosphorylated and therefore no hindrance to F-actin polymerization. When similar studies were carried out with Alexa Fluor 488 conjugated phalloidin, similar F-actin polymerization was observed only around the mycobacteria-containing phagosomes inside Cor1^-/-^ BMDM, not around the mycobacteria-containing phagosomes inside WT BMDM (**Figure 5D**). Fluorescence intensity calculations were next carried out, where intensity of Alexa Fluor 488 fluorescence corresponding to the presence of polymerized F-actin around the mycobacteria-containing phagosome was measured using a fixed line (denoted in red in **Figure 5D**). Such arbitrary fluorescence units that were obtained from 50 cells were averaged and plotted. It was observed that compared to mycobacteria infected WT BMDM, mycobacteria infected Cor1^-/-^ BMDM exhibited significantly higher levels of fluorescence intensity values (**Figure 5E**). This indicated that after 2 h of mycobacterial infection there was considerable amount of F-actin polymerization around the mycobacteria-containing phagosomes of Cor1^-/-^ BMDM alone (**Figure 5E**). When both WT and Cor1^-/-^ BMDM were pretreated with Slingshot Inhibitor D3 before mycobacterial infection, there was a considerable increase in F-actin polymerization around the phagosomes of WT BMDM macrophages in comparison to untreated but mycobacteria infected WT BMDM, but there was no significant increase in F-actin polymerization in the D3 pretreated Cor1^-/-^ BMDM upon mycobacterial infection compared to the untreated but mycobacteria infected Cor1^-/-^ BMDM (**Figure 5E**). From these data, it is evident that Cor1-induced increase of cAMP-mediated activation of Cof1 through its dephosphorylation by Slingshot is required to prevent F-actin polymerization at the vicinity of the mycobacteria-containing phagosome, which thereby prevents its fusion with the lysosome and allows mycobacterial survival.

### Coronin1 Activated Cofilin1 Also Hinders Phagosome Acidification

**Figure 6:**
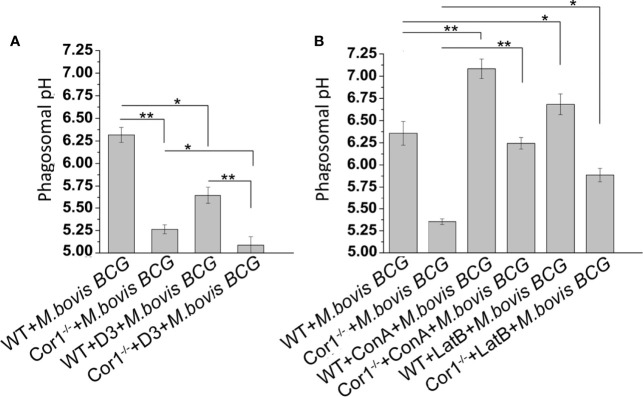
Phagosome pH was monitored using pHrodo in WT and Cor1^-/-^ BMDM upon being either **(A)** kept untreated or D3 pretreated before infection with *M. bovis BCG* for 3 h. **(B)** Similarly, WT or Cor1^-/-^ macrophages were either kept untreated or pretreated with either ConA or LatB before infection with *M. bovis* for 3 h, followed by measurement of phagosome pH. The phagosome pH values were obtained from the standard curve generated using pHrodo in different pH buffers. Data represents mean ± SEM; *p < 0.05, **p < 0.01.

Prevention of lysosomal delivery is essentially associated with inhibition of phagosome acidification (31). Cor1 mediated activation of Cofilin1, through the rise of intracellular cAMP, hinders lysosomal delivery of mycobacteria-containing phagosome. We asked whether it is solely due to inhibition of F-actin polymerization or does Cofilin1 prevent the acidification of mycobacteria-containing phagosomes? For this, mycobacteria were dual-labelled with pH-sensitive, pHrodo, and pH insensitive Alexa Fluor 488, followed by infection of macrophages and analysis of phagosome pH in a multimode reader. A pH standard curve (**Supplementary Figure 5**) was generated following the protocol stated in the *Materials and Methods* section. The pH of mycobacteria-containing phagosomes remains 6.4 after 3 h of infection, while in the case of Cor1^-/-^ BMDM, the mycobacteria-containing phagosome pH falls to 5.3 at a similar time point after infection (**Figure 6A**). In terms of phagosome acidification and fusion with lysosomes, this fall of pH to 5.3 is significantly high. Further pretreatment of WT BMDM with Slingshot Inhibitor D3 before infection resulted in acidification of the mycobacteria-containing phagosomes in 3 h post-infection. Hence, the pH falls from 6.4 to 5.6. In the case of the Cor1^-/-^ BMDM, D3 pretreatment prior to mycobacterial infection results in a further drop of phagosomal pH compared to untreated cells. The fall in pH in both WT and Cor1^-/-^ BMDM upon pretreatment with D3 prior to mycobacterial infection was similar to that observed in the case of the untreated cells. Additionally, pretreatment of macrophages with vacuolar H+-ATPase inhibitor Concanamycin A (ConA) maintains mycobacteria-containing phagosome pH above 7 for WT and above 6 for Cor1^-/-^ BMDM, respectively, which is significantly higher than the pH values of the untreated but infected cells (**Figure 6B**). This indicated that ConA, by inhibiting the vacuolar H+-ATPase, can prevent the pumping of H+ ions into the phagosome and thereby hinder phagosome acidification. To check if F-actin polymerization plays a role in phagosome acidification, next we pretreated macrophages with actin-depolymerizing agent Latrunculin B (LatB) before mycobacterial infection. After that, when phagosome pH was monitored (**Figure 6B**), it was observed that for LatB treated WT BMDM macrophages infected with mycobacteria, the phagosome pH rose to ~6.7 from ~6.3 for that of untreated WT BMDM macrophages infected with mycobacteria, while for LatB treated Cor1^-/-^ BMDM macrophages infected with mycobacteria the phagosome pH rose to ~5.9 from ~5.4 for that of untreated Cor1^-/-^ BMDM macrophages infected with mycobacteria. Thus, by inducing actin depolymerization, LatB could hinder the acidification of the phagosome as evident from a higher phagosome pH value upon LatB treatment. Therefore, the above data show that Cor1 activated Cof1 prevents phagosome acidification and thereby hinders phagosome-lysosome fusion. 

## Discussion

With the turn of every century, there has been a rise and fall of one pandemic or another. Tuberculosis continues to plague humanity and is a leading cause of mortality worldwide. The success of its causative agent, mycobacteria, lies in its ability to continuously adapt to its ever-changing environment, leading to several paradigm shifts in its pathogenesis (3). Previously, it was believed that mycobacteria could recruit the macrophage coat protein Cor1 on the phagosome membrane and thereby prevent its maturation. Recent studies show that in active TB, mycobacteria hinder the process of phagosome maturation until it can overexpress proteins that enable it to withstand acid stress of the lysosome (32). Contrary to this, perhaps when mycobacteria fail to hinder phagosome maturation, it secretes proteins that disrupt the phagosome membrane and allows the cytosolic escape of the infected mycobacteria. Once in the cytosol, mycobacteria engage the autophagic pathway, whereinafter autophagosomes and autophagolysosomes are formed gradually, thus providing time for mycobacteria to overexpress their acid-tolerant proteins (33).

Ensuing mycobacterial infection, macrophage Cor1 acts as the sentinel, and by being a cortical protein is present on the phagosome coat. Through its secretion of lipoamide dehydrogenase, mycobacteria can retain Cor1 on the phagosome membrane (23). Cor1-induced rise of intracellular Ca^2+^ upon mycobacterial infection causes the phosphatase calcineurin activation and thereby hinders phagosome maturation (16). However, this study did not explain how Cor1 directly or indirectly through calcineurin can delay phagosome maturation. It has also been shown that cAMP induces actin depolymerization at the vicinity of mycobacteria-containing phagosomes (21) thus preventing actin tracts from forming. Such actin tracts are required for the process of phagosome acidification by fusing with vacuolar H+-ATPase containing vesicles and also thereafter for fusing with lysosomes. However, this study did not state the varied source for this increasing cAMP concentration and the mechanism through which cAMP prevents actin polymerization at the vicinity of the phagosome. To answer these questions, we carried out our study where we first found that apart from the soluble adenylate cyclases secreted by mycobacteria, macrophages also contribute primarily toward the increasing intracellular cAMP concentration upon infection. This cAMP production is dependent on macrophage Cor1 as the same is absent in Cor1^-/-^ macrophages. Cor1 has been implicated in increased cAMP production upon stimulation of GPCRs (11). The intracellular cAMP level in the uninfected condition is not high. Still, it rises only upon mycobacterial infection, thus indicating that like GPCR signaling, mycobacterial infection and its concomitant recruitment and retention of the Cor1 coat on the phagosome membrane act as triggers for Cor1 induced cAMP production inside the macrophage. Such Cor1 mediated rise of intracellular cAMP levels can be prevented by pretreatment of cells with AC inhibitor KH7 prior to mycobacterial infection. This then leads to a Th2 to Th1 conversion and phagosome maturation to predominantly eliminate the intracellular mycobacteria. Intracellular cAMP levels are known to be increased by inhibition of PDE4 (34). Hence, pretreatment with PDE4 inhibitor Rolipram prior to mycobacterial infection, although generated a higher level of cAMP, was not sufficient to prevent phagosome maturation of *M. smeg* containing phagosomes, possibly due to the inability of *M. smeg* to retain Cor1 on its phagosome and thus lacking Cor1 mediated cAMP production at the vicinity of the phagosome. Additionally, mycobacterial infection is known to increase intracellular Ca^2+^ level in a Cor1 dependent manner at the vicinity of the phagosome membrane, and this increased Ca^2+^ after that activates the phosphatase calcineurin. Increased Ca^2+^ can also activate phospholipase C (PLC) and diacylglycerol, of which PLC is known to trigger cAMP production. So, whether a mycobacterial infection-induced increase in intracellular cAMP is generated directly and indirectly through Cor1 remains to be determined.

An increased intracellular cAMP would activate the cAMP/PKA pathway, which then would induce phosphorylation mediated activation of MAPK to induce IFNg secretion by T-cells upon mycobacterial infection of macrophages (35, 36). But *M. tb via* its LAM is known to activate SOCS and CISH proteins that dampen IFNg signaling inside mycobacteria infected macrophages. Hence, activated MAPK in *M. tb* infected cells fails to induce a Th1 response as compared to that in *M. smeg* infected cells. Hence, despite the MAPK activation in both *M. tb* and *M. smeg* infected cells, the former has means to prevent the Th1 response of the macrophages while the later exhibits the same.

We also observed that mycobacterial infection retains Cor1 on the phagosome membrane and induces its overexpression. The burning question that arose was why Cor1 expression would increase and, if so, why does immunofluorescence or immunoblotting analysis with increasing time of infection not exhibit this? We postulated that phagosome retained Cor1 gets degraded continuously, and to replenish this degrading Cor1, an increased expression of Cor1 was observed. Thus, Cor1 could be in a state of dynamic equilibrium where it is constantly getting degraded and replenished. Previous studies have shown that mycobacterial infection induces Cor1 mediated influx of Ca2+, which activates calcineurin, PLC, and DAG. The latter is known to activate PKC and activated PKC has been shown to delocalize Cor1 from the cortical surface by phosphorylating it at the C-terminus (37). PKC-mediated phosphorylation renders Cor1 monomeric, which then is scaffolded by RACK1 and shuttled by cargo carrier protein 14-3-3ζ (38). The monomeric Cor1 might be getting degraded in a condition where the macrophage is infected with mycobacteria, and semblance to this is observed when pretreatment with proteasomal inhibitor MG132 before infection exhibited an increase in Cor1 concentration. Additionally, studies of nascent protein expression could also indicate increased Cor1 expression with increasing infection time. The natural question would be if Cor1 is getting phosphorylated and degraded, and therefore to maintain its concentration around the phagosome, it must be overexpressed. Then what induces its overexpression? Our yet unpublished data show that calcineurin triggers this overexpression of Cor1 and thus maintains it in a dynamic equilibrium around the phagosome.

**Figure 7:**
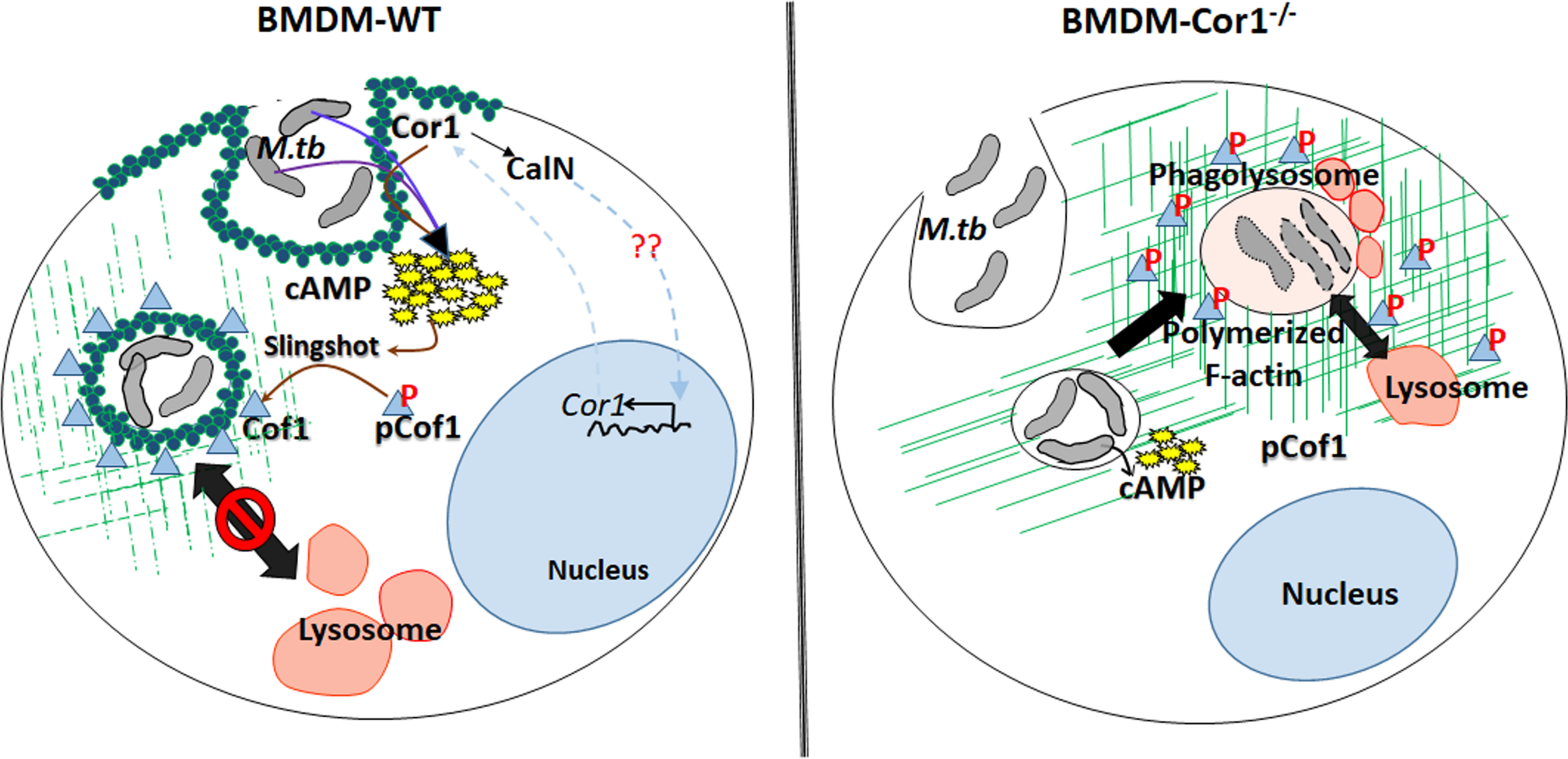
Schematic representation of Cor1-mediated increase of intracellular cAMP upon mycobacterial infection leading to activation of the phosphatase Slingshot and thereby dephosphorylation mediated activation of Cofilin1 to induce the depolymerization of F-actin to hinder the phagosome maturation and the absence of such pathway in Cor1^-/-^ macrophages.

This Cor1 induced, increased intracellular cAMP levels upon mycobacterial infection causes a concomitant activation of Cofilin 1 through its dephosphorylation by activated phosphatase Slingshot. Cof1 is known to prevent actin polymerization in its active dephosphorylated form, while cAMP has been shown to induce F-actin depolymerization at the vicinity of mycobacteria-containing phagosomes (**Figure 7**). So, it had to be determined whether the increased intracellular cAMP can directly influence the depolymerization of F-actin or the phosphatase, Slingshot-mediated activation of Cof1 causes F-actin depolymerization. Adenylate cyclase inhibitor-treated cells failed to dephosphorylate Cof1, thus indicating that increased intracellular cAMP was required for the dephosphorylation mediated activation of Cof1. Additionally, cells pretreated with Slingshot Inhibitor D3 also hindered Cof1 activation, thereby indicating the activation of Slingshot as the phosphatase for Cof1. Maturing phagosomes require continuously polymerizing F-actin tracts that bring them in proximity to vacuolar H^+^-ATPase containing vesicles for acidification and also in proximity to lysosomes to enable fusion and phagolysosome formation. Phagolysosome formation is hindered in the absence of such F-actin tracts. Previously, it was shown that increased intracellular cAMP could delay F-actin polymerization and thus prevent such tract formation (21), but the mechanism behind this was unknown. Here we show that cAMP, by activating Cof1, increases actin depolymerization, thereby hindering phagosome acidification and phagolysosome formation.

Thus, phagosome retained Cor1 increases the intracellular cAMP levels and thereby activates the phosphatase Slingshot, which then dephosphorylates Cof1 and activates it, such that it can depolymerize F-actin tracts presumably more at the vicinity of the phagosome and to some extent on other intracellular vesicles and organelles thus hindering phagosomal acidification as well as its maturation. Based on the present paradigm, the goal of infected mycobacteria is to delay phagosome acidification and its maturation to provide it with the required time to express the acid stress-tolerant proteins. Our study provides a mechanism through which mycobacteria achieve this. Mycobacteria retained Cor1 scaffold on the phagosome membrane is in a state of dynamic equilibrium, and it also induces F-actin depolymerization around the phagosome, thereby enabling very slow acidification where a semblance of F-actin brings in V-H+-ATPase to the phagosome membrane and allows its access in between the phase of degradation of phosphorylated Cor1 and replenishment by newly synthesized Cor1. This slow and gradual access would retard the acidification process considerably, thus enabling mycobacteria to get adapted to the acid stress, such that it can later thrive inside the phagolysosome.

Taken together, this study, for the first time, explicitly provides knowledge on the missing links in the context of Cor1 mediated hindrance to phagosome maturation and the role of cAMP in the context of retarded phagosome maturation. It brings to light the sequence of events that leads to the bumpy ride of mycobacteria-containing phagosome and thereby retards its maturation. It also provides an insight into the objective behind this event. Such studies on the causes and consequences of hindered phagosome maturation in the context of mycobacterial pathogenesis would enable us to curb the TB menace by purporting host-directed immunomodulatory therapeutics through the use of peptidomimetics.

## Data Availability Statement

The original contributions presented in the study are included in the article/**Supplementary Material**. Further inquiries can be directed to the corresponding author.

## Ethics Statement

The Institute Biosafety Committee approved the work, and for the use of *M. tb*, the work was permitted under the Biosafety Committee overseeing the use of the BSL3 facility at NJILOMD.

## Author Contributions

SS performed most of the experiments and wrote the manuscript. AH carried out some of the experiments. DG and SR helped SS execute some experiments and provided input toward the writing of the manuscript. AS provided the BSL3 facility and facilitated the execution of some experiments. SB planned the project and the course of the investigations and supervised manuscript writing. All authors contributed to the article and approved the submitted version.

## Funding

We would like to thank DBT, India (BT/RLF/Re-entry/33/2014), and DST-SERB (YSS/2015/000471 and CRG/2020/000748) for providing financial assistance for conducting the research. SS, AH, DG, and SR were provided financial assistance through a fellowship from IIT Kharagpur under MoE.

## Conflict of Interest

The authors declare that the research was conducted in the absence of any commercial or financial relationships that could be construed as a potential conflict of interest.

## Publisher’s Note

All claims expressed in this article are solely those of the authors and do not necessarily represent those of their affiliated organizations, or those of the publisher, the editors and the reviewers. Any product that may be evaluated in this article, or claim that may be made by its manufacturer, is not guaranteed or endorsed by the publisher.

## Supplementary Material

Supplementary Figure 1
**(A)** Analyzing the extent of infectivity corresponding to viability. WT-BMDM were infected with *M. tb* at indicated MOI followed by analysis of cell viability and extent of *M. tb* infection in these viable cells (n = 50). **(B)** Competitive ELISA-based measurement of cAMP production of BMDM at indicated MOI of *M. tb* infection at indicated time points. **(C)** Extent of phagolysosome formation in live *M. tb, M. bovis BCG, M. smeg*, heat-killed *M. tb*, and heat-killed *M. bovis BCG* infected BMDM after 3 h of infection, without and after pretreatment with KH7 or Rolipram (n = 50). Data represents mean ± SEM; **p* < 0.05, ***p* < 0.01, NS, non significant. **(D)** Immunofluorescence analysis of mycobacteria-infected macrophages. BMDM were infected with live *M. smeg* or *M. tb* for indicated time points and thereafter stained for mycobacteria (green), lysosomes (LAMP2 in red), and Cor1 (blue). In case of *M. smeg*, Cor1 fails to form a scaffold around the phagosome, which leads to phagosome-lysosome fusion at 3 h, while for *M. tb-*infected macrophages, the Cor1 scaffold around the phagosome hinders its maturation and hence it does not fuse with lysosomes (scale: 10 μm). **(E)** Extent of phagolysosome formation in *M. smeg* or *M. tb* infected BMDM after 3 h of infection and without and with pretreatment with KH7 or Rolipram (n = 100). Data represents mean of triplicates ± SEM.

Supplementary Figure 2 Immunoblotting of WT, Cor1^-/-^, and c-myc tagged Cor1 transfected Cor1^-/-^ BMDM with anti-Cor1 and anti-c-myc antibodies exhibits expression of Cor1 in WT and Cor1^-/-^ transfected with Cor1 and band corresponding to c-myc was observed only in Cor1 expressing Cor1^-/-^ cells, indicating successful transfection. b-tub was used as loading control.

Supplementary Figure 3
**(A)** Immunoblot of Cor1 from BMDM whole-cell lysates that were either kept uninfected or infected with live or heat-killed *M. tb*, *M. bovis BCG*, or live *M. smeg* did not exhibit the apparent increase of Cor1 expression upon infection. **(B)** Immunoblot and its corresponding densitometry analysis for Cor1 expression in WT-BMDM infected with *M. tb* after being pretreated and in the presence of MG132. Gradual increase in Cor1 expression normalized over that of b-tub control is observed (n = 3). Data represents mean ± SEM; **p* < 0.05, ***p* < 0.01.

Supplementary Figure 4 qPCR analysis of Th1/Th2 response in BMDMs upon infection with *M. smeg* or *M. tb*, without and with pretreatment with KH7 and Rolipram. Based on the overexpression of **(A)** TNFa and **(C)** iNOS2, Th1 response was evident in *M. smeg* infected macrophages without or with KH7 or Rolipram pretreatment and partly in KH7 pretreated and *M. tb* infected macrophages. Overexpression of **(B)** IL-10 and **(D)** Arg1 in *M. tb* infected cells without and with Rolipram pretreatment is indicative of Th2 response in these macrophages.

Supplementary Figure 5 Mycobacteria labelled with pH-sensitive pHrodo and pH-insensitive Alexa Fluor 488 were grown in 7H9 buffer of pH 4.5-8.5. The ratio of pHrodo to Alexa Fluor 488 fluorescence in different buffers was plotted to obtain the pH standard curve and used to measure phagosomal pH upon mycobacterial infection (n = 3).
