# Supplementary figures and images for: A Bumpy Ride of Mycobacterial Phagosome Maturation: Roleplay of Coronin1 Through Cofilin1 and cAMP
Source: Front Immunol. 2021 Sep 23;12:687044. doi: 10.3389/fimmu.2021.687044 (PMC8495260; doi:10.3389/fimmu.2021.687044)

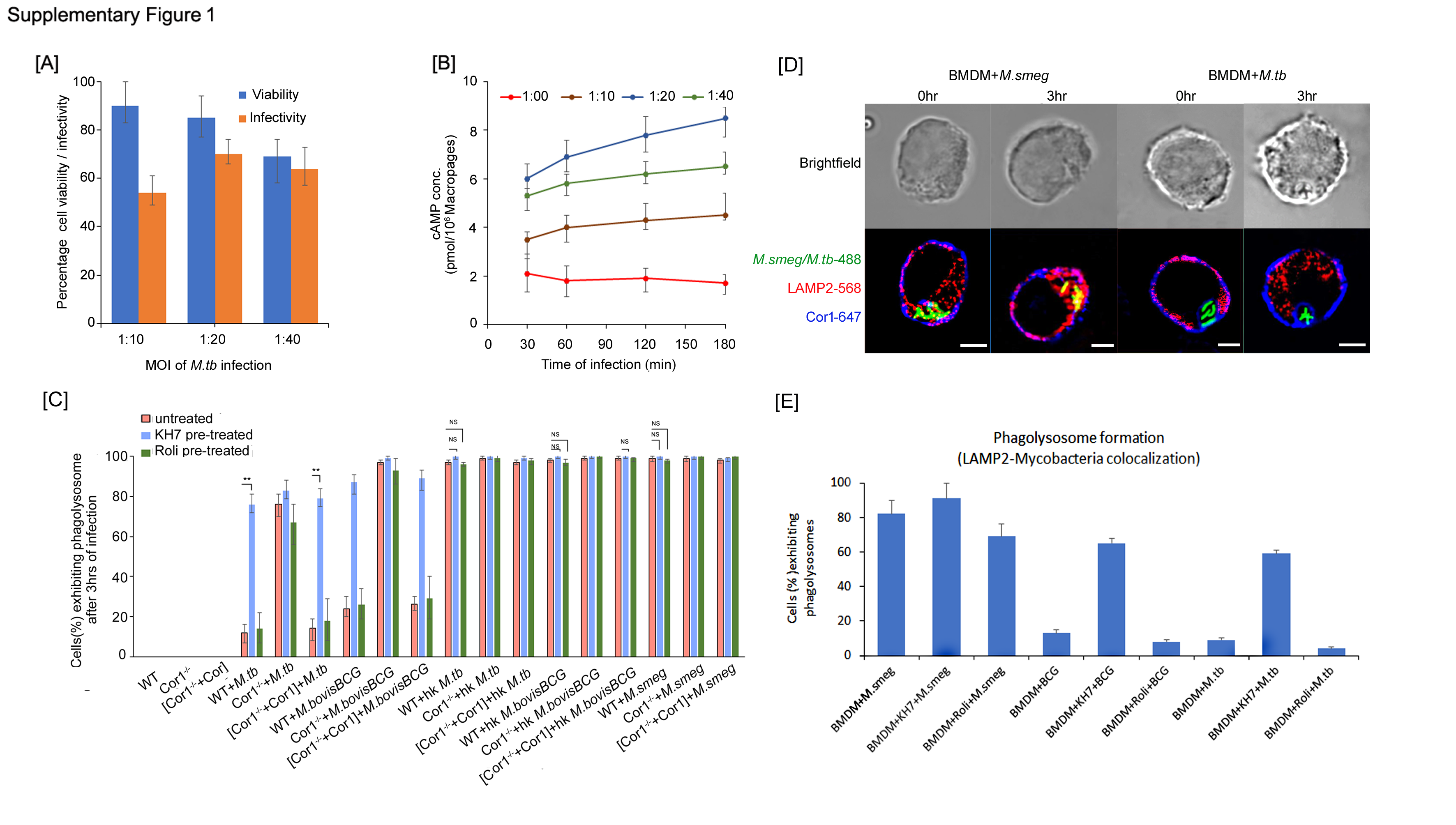

Supplement: Supplementary Figure 1 — (A) Analyzing the extent of infectivity corresponding to viability. WT-BMDM were infected with M. tb at indicated MOI followed by analysis of cell viability and extent of M. tb infection in these viable cells (n = 50). (B) Competitive ELISA-based measurement of cAMP production of BMDM at indicated MOI of M. tb infection at indicated time points. (C) Extent of phagolysosome formation in live M. tb, M. bovis BCG, M. smeg, heat-killed M. tb, and heat-killed M. bovis BCG infected BMDM after 3 h of infection, without and after pretreatment with KH7 or Rolipram (n = 50). Data represents mean ± SEM; *p < 0.05, **p < 0.01, NS, non significant. (D) Immunofluorescence analysis of mycobacteria-infected macrophages. BMDM were infected with live M. smeg or M. tb for indicated time points and thereafter stained for mycobacteria (green), lysosomes (LAMP2 in red), and Cor1 (blue). In case of M. smeg, Cor1 fails to form a scaffold around the phagosome, which leads to phagosome-lysosome fusion at 3 h, while for M. tb-infected macrophages, the Cor1 scaffold around the phagosome hinders its maturation and hence it does not fuse with lysosomes (scale: 10 μm). (E) Extent of phagolysosome formation in M. smeg or M. tb infected BMDM after 3 h of infection and without and with pretreatment with KH7 or Rolipram (n = 100). Data represents mean of triplicates ± SEM. [file Image_1.tif]

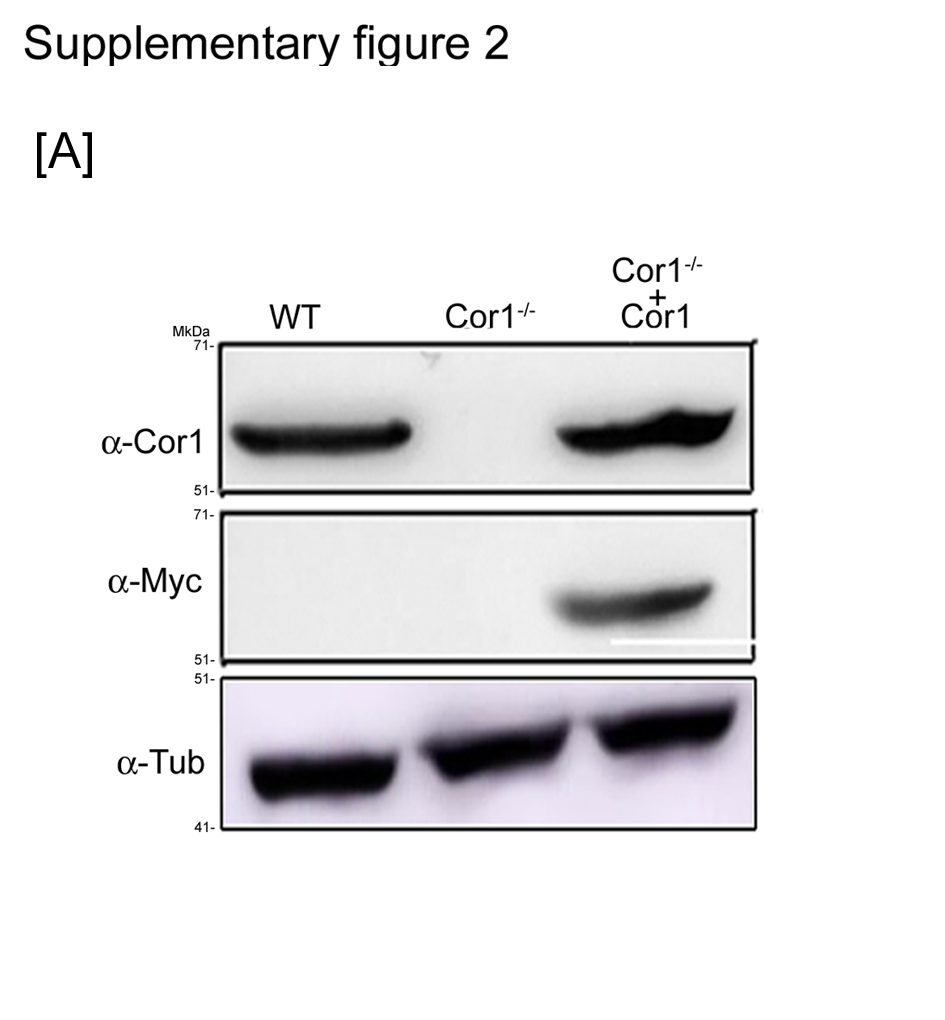

Supplement: Supplementary Figure 2 — Immunoblotting of WT, Cor1-/-, and c-myc tagged Cor1 transfected Cor1-/- BMDM with anti-Cor1 and anti-c-myc antibodies exhibits expression of Cor1 in WT and Cor1-/- transfected with Cor1 and band corresponding to c-myc was observed only in Cor1 expressing Cor1-/- cells, indicating successful transfection. b-tub was used as loading control. [file Image_2.tif]

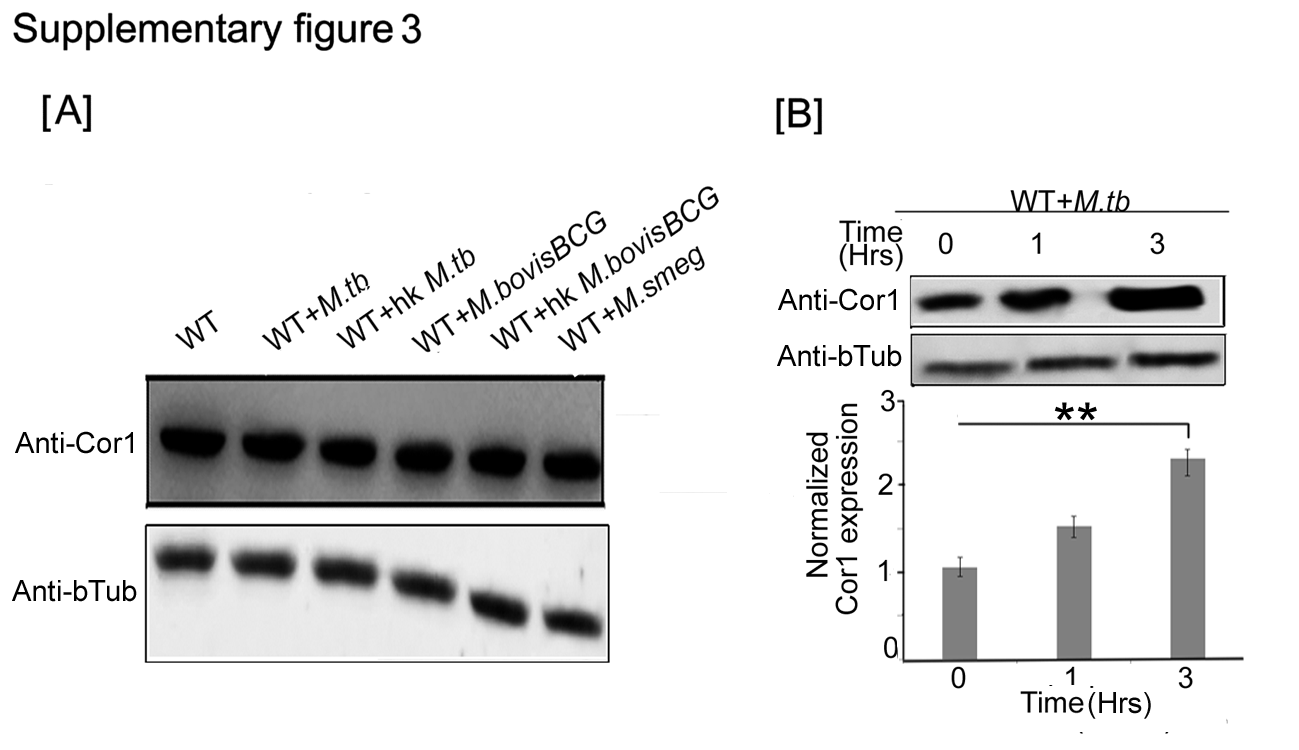

Supplement: Supplementary Figure 3 — (A) Immunoblot of Cor1 from BMDM whole-cell lysates that were either kept uninfected or infected with live or heat-killed M. tb, M. bovis BCG, or live M. smeg did not exhibit the apparent increase of Cor1 expression upon infection. (B) Immunoblot and its corresponding densitometry analysis for Cor1 expression in WT-BMDM infected with M. tb after being pretreated and in the presence of MG132. Gradual increase in Cor1 expression normalized over that of b-tub control is observed (n = 3). Data represents mean ± SEM; *p < 0.05, **p < 0.01. [file Image_3.tif]

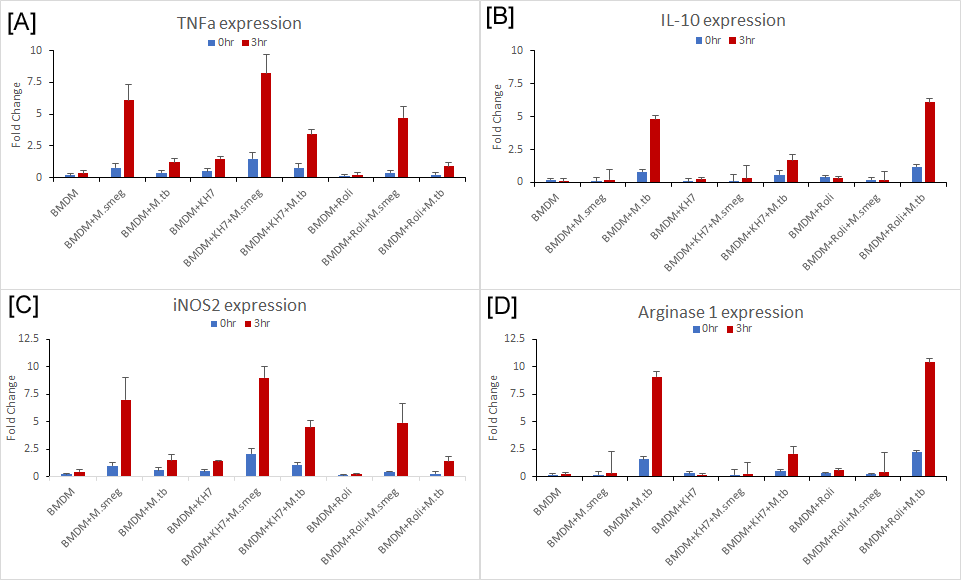

Supplement: Supplementary Figure 4 — qPCR analysis of Th1/Th2 response in BMDMs upon infection with M. smeg or M. tb, without and with pretreatment with KH7 and Rolipram. Based on the overexpression of (A) TNFa and (C) iNOS2, Th1 response was evident in M. smeg infected macrophages without or with KH7 or Rolipram pretreatment and partly in KH7 pretreated and M. tb infected macrophages. Overexpression of (B) IL-10 and (D) Arg1 in M. tb infected cells without and with Rolipram pretreatment is indicative of Th2 response in these macrophages. [file Image_4.tif]

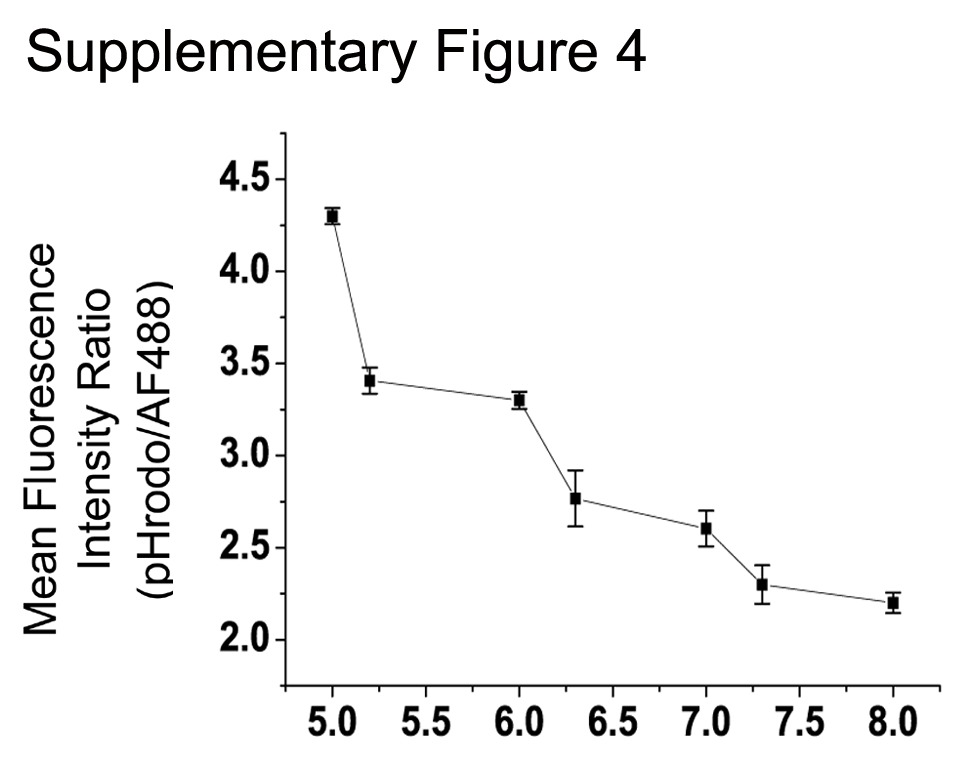

Supplement: Supplementary Figure 5 — Mycobacteria labelled with pH-sensitive pHrodo and pH-insensitive Alexa Fluor 488 were grown in 7H9 buffer of pH 4.5-8.5. The ratio of pHrodo to Alexa Fluor 488 fluorescence in different buffers was plotted to obtain the pH standard curve and used to measure phagosomal pH upon mycobacterial infection (n = 3). [file Image_5.tif]
